# Treatment-associated network dynamics in patients with globus sensations: a proof-of-concept study

**DOI:** 10.1038/s41598-023-42186-y

**Published:** 2023-09-20

**Authors:** Marina N. Imperiale, Roselind Lieb, Gunther Meinlschmidt

**Affiliations:** 1 Division of Clinical Psychology and Epidemiology, Department of Psychology, University of Basel, Missionsstrasse 62a, 4055 Basel, Switzerland; 2 Department of Clinical Psychology and Cognitive Behavioral Therapy, International Psychoanalytic University (IPU) Berlin, Stromstrasse 1, 10555 Berlin, Germany; 3 Department of Digital and Blended Psychosomatics and Psychotherapy, Psychosomatic Medicine, University Hospital Basel and University of Basel, Hebelstrasse 2, 4031 Basel, Switzerland

**Keywords:** Psychology, Human behaviour, Health care

## Abstract

In this proof-of-concept study, we used a systems perspective to conceptualize and investigate treatment-related dynamics (temporal and cross-sectional associations) of symptoms and elements related to the manifestation of a common functional somatic syndrome (FSS), Globus Sensations (GS). We analyzed data from 100 patients (*M* = 47.1 years, *SD* = 14.4 years; 64% female) with GS who received eight sessions of group psychotherapy in the context of a randomized controlled trial (RCT). Symptoms and elements were assessed after each treatment session. We applied a multilevel graphical vector-autoregression (ml GVAR) model approach resulting in three separate, complementary networks (temporal, contemporaneous, and between-subject) for an affective, cognitive, and behavioral dimension, respectively. GS were not temporally associated with any affective, cognitive, and behavioral elements. Temporally, catastrophizing cognitions predicted bodily weakness (r = 0.14, *p* < 0.01, 95% confidence interval (CI) [0.04–0.23]) and GS predicted somatic distress (r = 0.18, *p* < 0.05, 95% CI [0.04–0.33]). Potential causal pathways between catastrophizing cognitions and bodily weakness as well as GS and somatic distress may reflect treatment-related temporal change processes in patients with GS. Our study illustrates how dynamic NA can be used in the context of outcome research.

Functional somatic syndromes (FSS), including Globus Sensations (GS), are among the most prevalent disorders in the adult population^1,2^. FSS are best characterized by medically unexplained symptoms (MUS)^3^ and although the classification of FSS in general is controversial^4^, considerable overlap with the 5th edition of the Diagnostic and Statistical Manual (DSM-IV)^5^ somatic symptom disorder (SSD) is expected^4^. GS, more commonly known as the feeling of a “lump in throat”, is a FSS with lifetime prevalence reports of 9.1–21.5%^6,7^. The exact pathophysiology underlying GS is still unclear^8^ and a current literature review^9^ suggests a complex and multiform etiology. Both somatic (i.e., gastroesophageal reflux disorder, laryngopharyngeal reflux, esophageal motor disorders, and upper esophageal sphincter abnormalities) and psychological factors, including stress, have been suggested to contribute to GS. In particular, psychological factors such as negative affect and dysfunctional cognitive-perceptual processes may play a role in the development and maintenance of FSS and MUS more generally^10–13^, suggesting a potential role of these factors in GS. In sum, etiology of GS is unclear with current literature pointing towards a contribution of both somatic and psychological factors to the etiology of GS.

Given the lack of uniformity regarding the etiology of GS, no standard treatment guideline exists. Previous studies provide support that psychotherapy [e.g., cognitive-behavioral therapy (CBT), mindfulness-based interventions, or progressive muscle relaxation (PMR)] may be effective in the treatment of FSS and MUS^4,14–16^, suggesting that psychotherapy may also be effective in the treatment of GS. In fact, some studies have demonstrated efficacy of CBT in the treatment of GS^8,14,17^ and one case-series report provides evidence that treatment focusing on stress reduction and relaxation can provide substantial improvement of GS^18^. However, despite the high prevalence of GS, patients largely have unmet treatment needs^10^ due to lack of data from randomized clinical trials and unclarity regarding therapeutic mechanisms. In sum, GS are highly prevalent^7^ but often undertreated due to lack of evidence concerning efficacy of treatment and therapeutic mechanisms, as well as factors related to the development and maintenance of symptoms.

Previously, outcome research in psychotherapy has focused mainly on absence versus presence of a disorder or the severity level of a disorder. Disorders are viewed as an underlying entity (i.e., as latent variable) causing symptoms and covariation between symptoms is assumed due to the common cause (e.g., disorder). As such, symptoms are indicators of the latent construct and are summarized into a total score reflecting the absence/presence or severity of the disorder. Although sum score changes may be relevant in demonstrating change (i.e., treatment compared to no treatment leads to therapeutic change), it does not shed light on how an intervention led to change. The current trend towards evidence-based therapy calls for analytical approaches that can provide insight into mechanisms of change^19^. Novel methodologies such as dynamic network analysis (NA) that go beyond the limitations of contemporary approaches to understand the process of change (i.e. linear mediation) have recently been adopted in the field of psychotherapy research and are promising with regard to modeling complex processes such as therapeutic change^19^. The network approach was introduced a little over a decade ago and offers a conceptual framework using a systems perspective to study psychological processes. A network model consists of elements or “nodes”, oftentimes represented by symptoms. However, nodes can also represent other elements such as syndromic features of a disorder, contextual features, or indicators of change mechanisms (e.g., psychological flexibility). “Edges” represent the statistical relationship between the nodes. Edges are non-observable and are estimated from data. Most of the research done with network models in psychology has focused on cross-sectional data which results in undirected network models^20,21^. Cross-sectional studies offer information concerning temporally undetermined relationships between elements. However, they come with an important limitation, as they are not able to uncover temporal relationships between elements, which can be key to specific research queries particularly related to mechanisms of change. Dynamic network models, however, provide a methodological framework enabling a systems approach towards elucidating the dynamics (i.e., temporal and cross-sectional associations) between elements relevant in a particular disorder^19^. Network models are data-driven, can include a wide range of elements associated with assumed mechanisms of change (i.e., in the case of GS, affective, cognitive, and behavioral elements associated with its manifestation), and focus on functional relationships between elements. The theoretical framework of NA and specifically the focus on functional relationships between elements is especially close to clinical practice, where the goal is often to uncover functional patterns that lead to symptom expression. In sum, the trend towards evidence-based treatments calls for methodological approaches such as dynamic NA, which may enable the examination of processes of therapeutic change.

In the present study, we applied the network approach and conceptualize treatment-related dynamics between elements that have been linked to the manifestation of FSS^10–13^ in patients with GS. Specifically, we investigate temporal and cross-sectional associations between affective, cognitive, and behavioral elements that may play a role in the manifestation of GS over the course of treatment. Our aim was to elucidate the dynamic interplay between elements specific to these three dimensions over the course of treatment. By doing so we address the gap in research by demonstrating how dynamic NA techniques may be used to inform the development of evidence-based treatment approaches in patients with GS.

## Results

### Descriptive statistics

**Figure 1:**
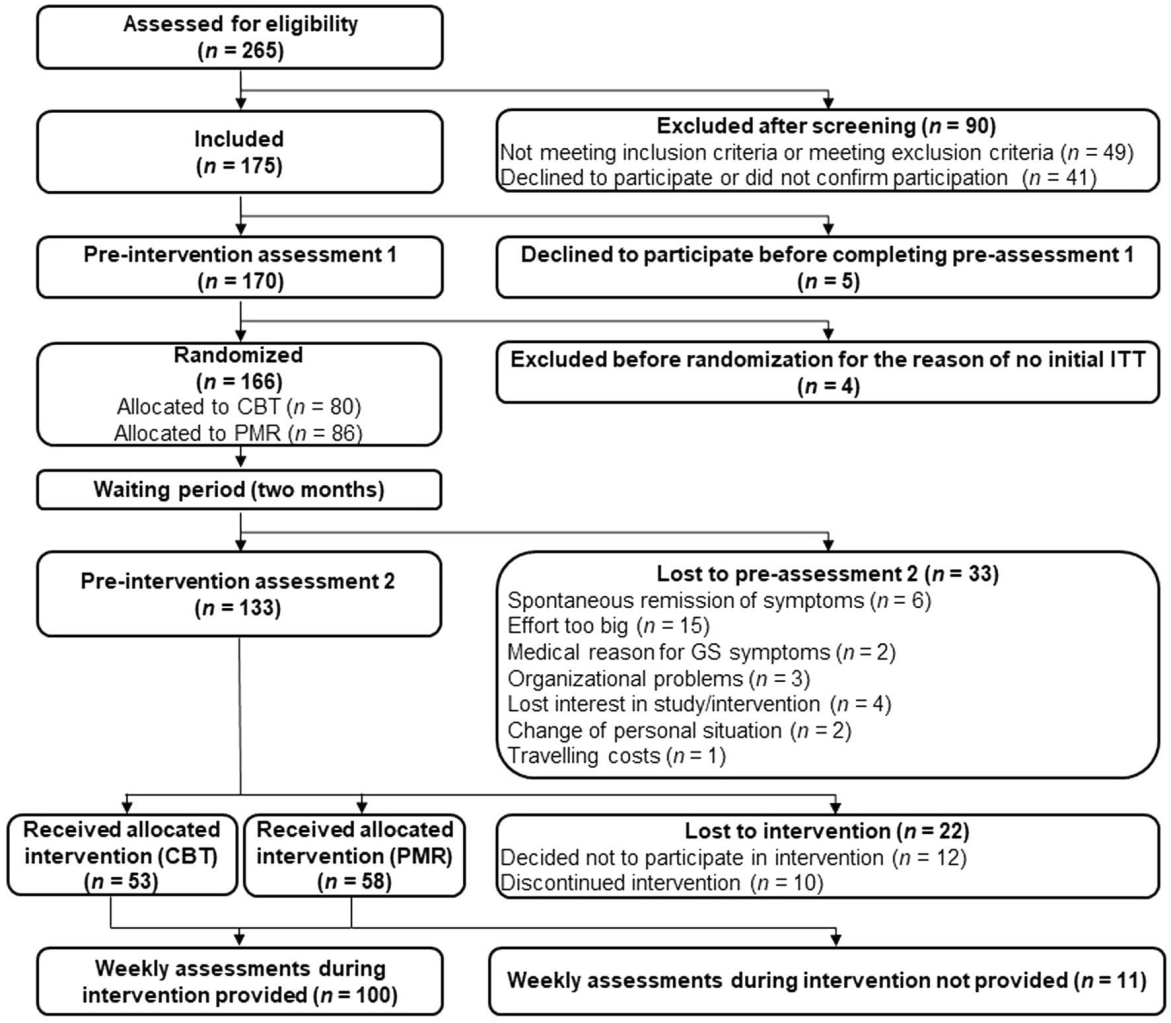
Flow Chart. *Note.* This figure depicts the flow chart of the study procedure. *CBT* Cognitive Behavioural Therapy. *GS* Globus Sensations. *ITT* Intention-to-treat. *PMR* Progressive Muscle Relaxation.

The analyses are based on data from *N* = 100 patients. We excluded *n* = 4 patients due to lack of at least one intra-intervention assessment. We described the sociodemographic characteristics of the sample in Table 1; Fig. 1.

**Table 1:** Sociodemographic characteristics of the sample.

N	Total sample
	100
Age (years)	
Mean	47.1
Standard deviation	14.4
Sex	
Male	36
Female	64
Comorbidity^a^	
With current depression or anxiety	33
Without current depression or anxiety	67
Educational level	
Mandatory education or less	2
Apprenticeship	32
High school graduation	7
Professional training	23
University	34
Unknown	2
Marital status	
Single	17
In a relationship	28
Married	42
Separated/divorced/widowed	12
Unknown	1
Living situation	
Lives alone	26
Lives with at least one other person	74
Employment	
Currently employed	74
Currently unemployed	25
Unknown	1
Net household income per month (CHF)	
Less than 4500	37
4500–7500	24
7500–11,250	14
11,250–15,000	8
Above 15,000	1
Declined answer/Unknown	16

This table describes the sociodemographic characteristics of the total sample. CHF: Swiss franc. CIDI: The World Health Organization Composite International Diagnostic Interview.

^a^Comorbidity was only assessed with respect to Depression or Anxiety Disorder. Current refers to a past 12 months CIDI-diagnosis.

### Network estimation and visualization

**Figure 2:**
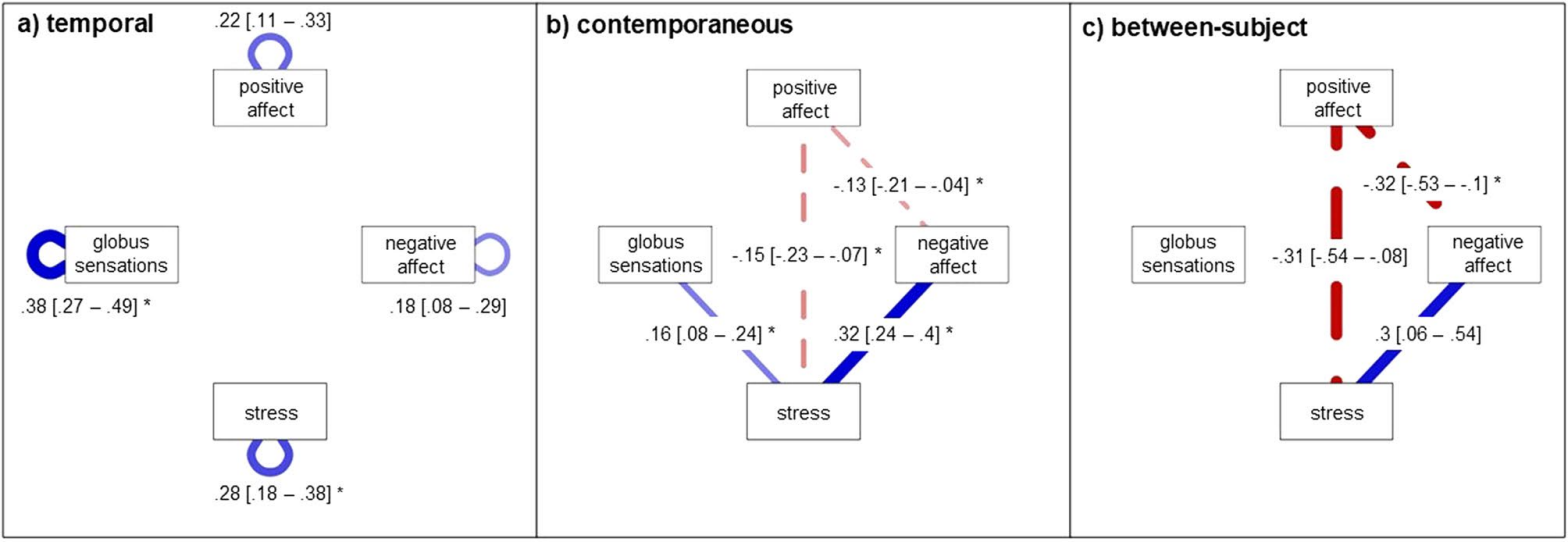
Associations Between Affective Elements and GS. *Note.* Temporal, contemporaneous and between-subject network model of affective elements and GS. Edges represent autocorrelations (temporal network) and partial correlations. 95% CIs are provided in brackets [] next to the partial correlation for each edge. Positive associations are depicted in blue, negative associations are dashed and in red. Maximum edge strength is set to the strongest edge identified across all three networks so that edges are visually comparable. *CI* Confidence interval. *GS* Globus Sensations. * indicates stable edges, i.e., edges that were included in at least 50% of the bootstrapped samples.

The baseline multilevel graphical vector autoregression model (ml GVAR) for the first analysis showed close fit (CFI = 0.8; TLI = 0.81; RMSEA = 0.09, 95% confidence intervals (CI) [0.081–0.1]; BIC = 5416.47). The pruned model showed better fit (CFI = 0.8; TLI = 0.81; RMSEA = 0.089 with 95% CI [0.08–0.098]; BIC = 5356.11), performing slightly better than the baseline model (∆BIC = 60.36). The pruned model using the step-up search did not improve fit indices compared to the pruned model without the step-up search (CFI = 0.8; TLI = 0.81; RMSEA = 0.089 with 95% CI [0.08–0.098]; BIC = 5356.11). Figure 2 depicts the temporal, contemporaneous, and between-subject networks of the first analysis: the association between symptoms of the affective dimension and GS. The temporal network describes how each variable after treatment session t predicts itself and all other variables at treatment session t + 1. All variables predicted themselves a week later. Importantly, the temporal network showed no other relationships, that is none of the variables predicted any of the other variables at the next time point. The contemporaneous network describes how variables are associated with each other within the same time frame after adjusting for temporal associations. We use Cohen’s conventions ^22^ to interpret effect size: (+ /−) 0.1 = small/weak, (+ /−) 0.3 = moderate, and (+ /−) 0.5 = large. Here we found a small positive partial correlation between GS and stress and a moderate positive partial correlation between NegA and stress. PosA was weakly negatively associated with NegA and stress. The between-subject network describes how variables on average are related to each other. In contrast to the contemporaneous network, modelling weekly fluctuations between elements, the between-subject network models the long-term stable mean differences between subjects. Comparable to the contemporaneous network, PosA was moderately negatively associated with NegA and stress. NegA and stress were moderately positively associated. In contrast to the contemporaneous network, there was no association between GS and other affect-related constructs.

**Figure 3:**
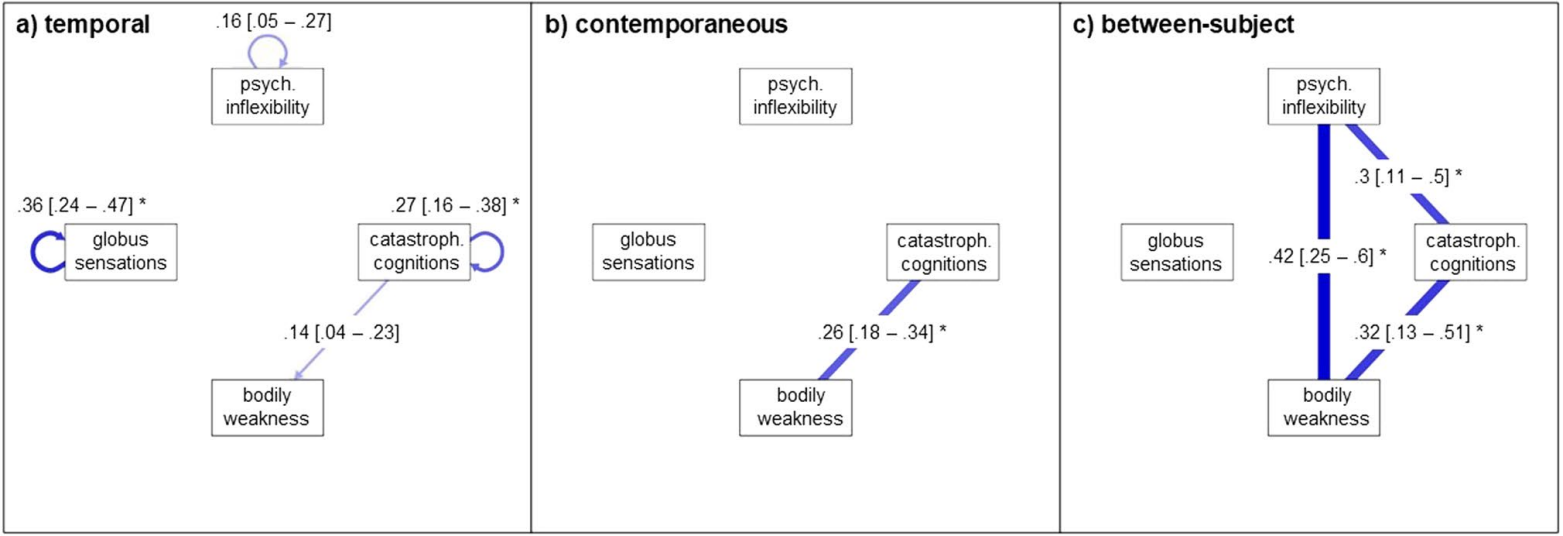
Associations Between Cognitive Elements and GS. *Note.* Temporal, contemporaneous and between-subject network model of cognitive elements and GS. Edges represent autocorrelations (temporal network) and partial correlations. 95% CIs are provided in brackets [] next to the partial correlation for each edge. Positive associations are depicted in blue, negative associations are dashed and in red. Maximum edge strength is set to the strongest edge identified across all three networks so that edges are visually comparable. *CI* Confidence interval. *GS* Globus Sensations. * indicates stable edges, i.e., edges that were included in at least 50% of the bootstrapped samples.

The baseline ml GVAR model for the second analysis showed close to acceptable fit (CFI = 0.89; TLI = 0.9; RMSEA = 0.08 with 95% CI [0.08–0.094]; BIC = 4070.6). The pruned model also showed close fit (CFI = 0.88; TLI = 0.89; RMSEA = 0.09 with 95% CI [0.08–0.1]; BIC = 4017.88), performing slightly better than the baseline model (∆BIC = 52.72). The pruned model using the step-up search performed slightly better than the pruned model alone (CFI = 0.89; TLI = 0.9; RMSEA = 0.08 with 95% CI [0.08–0.09]; BIC = 4011.29) with ∆BIC = 6.59. Figure 3 depicts the temporal, contemporaneous, and between-subject networks of the second analysis: the association between dysfunctional cognitions and GS. Again, almost all variables except for bodily weakness (i.e., having a negative self-concept of being weak and not being able to tolerate stress) predicted themselves a week later. The temporal network also revealed a positive directed correlation from catastrophizing cognitions to bodily weakness. The contemporaneous network showed a small positive partial correlation catastrophizing cognitions and bodily weakness. The between-subject network showed a moderate positive partial correlation between psychological inflexibility and bodily weakness. Additionally, average scores of psychological inflexibility were moderately positively associated with average scores for catastrophizing cognitions. Importantly, all associations between psychological inflexibility and other elements are only found in the between-subject networks. The between-subject network also showed moderate positive partial correlations between catastrophizing cognitions and bodily weakness.

**Figure 4:**
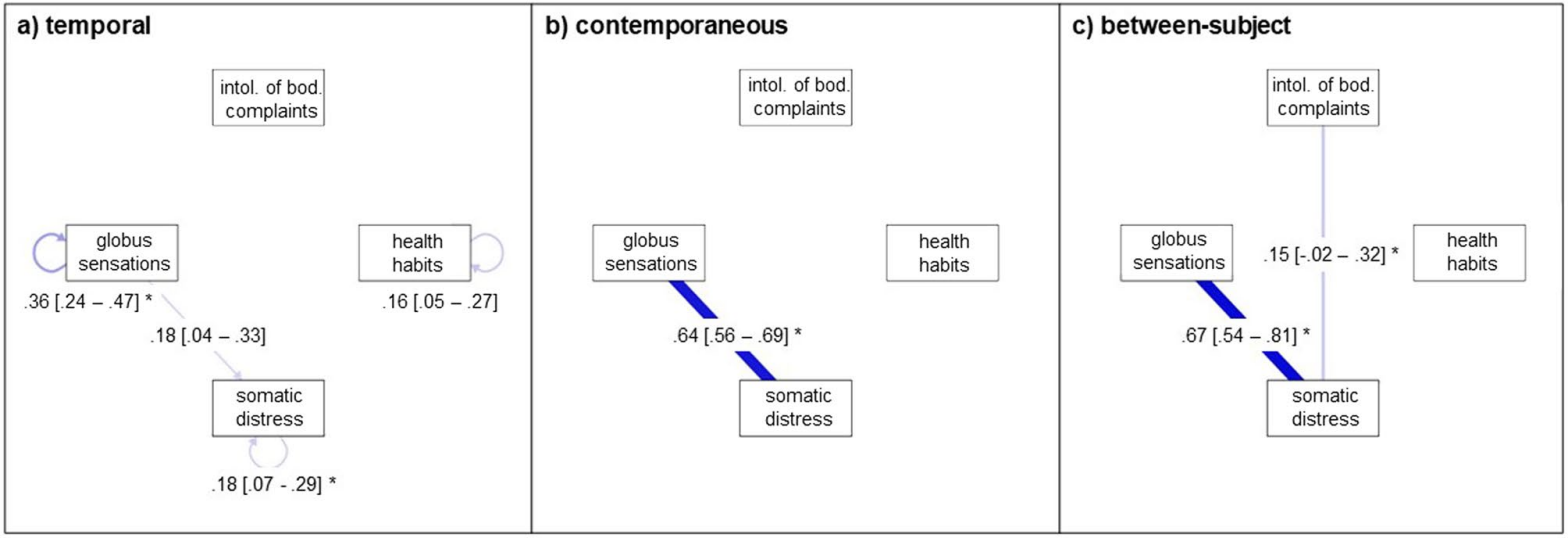
Associations Between Behavioral Elements and GS. *Note.* Temporal, contemporaneous and between-subject network model of behavioral elements and GS. Edges represent autocorrelations (temporal network) and partial correlations. 95% CIs are provided in brackets [] next to the partial correlation for each edge. Positive associations are depicted in blue, negative associations are dashed and in red. Maximum edge strength is set to the strongest edge identified across all three networks so that edges are visually comparable. *CI* Confidence interval. *GS* Globus Sensations. * indicates stable edges, i.e., edges that were included in at least 50% of the bootstrapped samples.

The baseline ml GVAR model for the third analysis showed close to acceptable fit (CFI = 0.85; TLI = 0.86; RMSEA = 0.087 with 95% CI [0.078–0.097]; BIC = 4746.27). The pruned model also showed close to acceptable fit (CFI = 0.85; TLI = 0.86; RMSEA = 0.087 with 95% CI [0.078–0.096]; BIC = 4681.85). It performed slightly better than the baseline model (∆BIC = 64.42). The pruned model using the step-up search performed minimally better than the pruned model alone (CFI = 0.85; TLI = 0.86; RMSEA = 0.086 with 95% CI [0.077–0.095]; BIC = 4676.73) with ∆BIC = 5.12. Figure 4 depicts the temporal, contemporaneous, and between-subject networks of the third analysis: the association between symptoms and cognitions related to the behavioral dimension and GS. Similar to the previous temporal networks, all of the variables except intolerance of bodily complaints had positive autocorrelations meaning they positively predicted themselves at the subsequent assessment point. The temporal network also showed that GS preceded somatic distress, i.e., higher levels of Globus-specific symptoms at a previous measurement occasion predicted higher levels of somatic distress at the next measurement occasion. The contemporaneous network showed a strong positive partial correlation between GS and somatic distress. We found no other associations in the contemporaneous network. The between-subject network presented a strong positive partial correlation between GS and somatic distress. The association between somatic distress and intolerance of bodily complaints was non-significant.

To examine the stability of the estimated network parameters, we created 1000 bootstrap samples, where 25% of the original data was dropped each time. We estimated the temporal, contemporaneous, and between-subject networks with the bootstrap samples, using the model search algorithm applied in the main analyses and calculated the frequencies with which each edge was included in the 1000 estimations. We provide the frequencies for each edge in Supplementary Tables S10–S12. Edges included in at least 50% of the bootstrapped samples were considered stable. Stable edges are marked in Figs. 2, 3, and 4. We found high stability in the contemporaneous networks of all three analyses as well as in the between-subject networks for the for the analysis examining the association between GS and cognitive elements and the analysis examining the association between GS and behavioral elements. Furthermore, we found moderate stability in the temporal networks for all three analyses and between-subject network for the analysis examining the association between GS and affective elements, suggesting respective edges should be interpreted with caution.

## Discussion

In this study, we examined temporal and cross-sectional associations between key elements related to the manifestation of FSS in patients with GS over the course of an 8-week group psychotherapy. Using a systems-perspective to conceptualize treatment-related dynamics, we applied dynamic NA methods and estimated three distinct, complementary networks (temporal, contemporaneous, and between-subject) for symptoms and elements of affective, cognitive, and behavioral dimensions, respectively, to examine the interplay of key elements in each dimension while distinguishing between within-subject and between-subject effects. Our aim was to evaluate the dynamics between symptoms and affect, dysfunctional cognitions, and behavioral-related elements over the course of treatment.

There are six key findings of our study: First, temporal networks indicated that GS and affective, cognitive, and behavioral elements were relatively stable over short time periods (i.e., one week) in a treatment context. Second, GS were not temporally predicted by any other elements. Third, we found associations between several dysfunctional cognitions. Notably, catastrophizing cognitions preceded bodily weakness and may indicate a potential causal pathway. In addition, catastrophizing cognitions and bodily weakness fluctuated on a weekly basis and average levels of these cognitions were positively associated with each other. Furthermore, psychological inflexibility showed strong associations on a between-subject level with regard to catastrophizing cognitions and bodily weakness. Fourth, weekly fluctuations of GS were associated with weekly fluctuations of stress. Fifth, GS preceded somatic distress. Sixth, we provided evidence regarding feasibility of dynamic NA in the context of outcome research.

Currently, various psychotherapeutic approaches for management of FSS are recommended including CBT and PMR. The therapeutic rationale of these non-pharmacological interventions is primarily to increase overall functioning and coping^4^, although psychotherapeutic interventions have also shown positive effects on symptom severity^4,23^. Our finding that GS were not predicted by elements assumed to be related to the manifestation of FSS suggests that a direct impact of these elements on changes in symptom expression does not occur within the timeframe of a week. Furthermore, except for somatic distress, elements were not related to GS in any of the networks, which suggests that changes in symptom expression may be a result of other therapeutic processes or that GS-specific symptoms were relatively stable in the context of treatment and therefore did not show any associations with elements except for somatic distress. Overall, psychotherapeutic interventions for somatoform disorders have shown inconclusive evidence regarding the outcome symptom severity^24^ and those that found reduction of symptom severity reported small effect sizes^24,25^. Our findings emphasize that therapeutic interventions may not be associated with substantial changes in symptom expression within the timeframe of a week. Instead subtle relationship dynamics between elements, especially cognitive elements were revealed, which might not necessarily align with substantial therapeutic progress. Furthermore, changes in symptom expression may be independent from the affective, cognitive, and behavioral elements included in our networks. Additionally, it is possible that the spacing of the assessments (i.e., weekly) and the choice of elements may have affected the associations we found. However, we did find interesting associations between other elements that may represent therapeutic processes in relation to overall functioning and coping as well as common comorbidities of patients with GS. Our finding regarding a potential causal pathway between the dysfunctional cognitive attributional style referred to as catastrophizing cognitions, characterized by the presence of a very strict concept of health in which one must be relatively symptom free to be healthy and bodily weakness, which refers to a negative self-concept of feeling weak and unable to tolerate stress, represents a novel demonstration of the impact of a dysfunctional cognitive style on one’s self concept. The association between catastrophizing cognitions and bodily weakness was consistent in the contemporaneous and between-subject networks. Previously, it has been suggested that catastrophizing cognitions may contribute to the personal belief of being powerless^12,26^. Since feeling powerless is one aspect of bodily weakness, this finding provides further support for a causal relationship. However, our model fit indices were weak and results from the bootstrapped samples indicated limited stability. Hence the edge between catastrophizing cognitions and bodily weakness should be interpreted with caution. Therefore our findings may not be robust. More research is needed to replicate these findings. Interestingly, bodily weakness did not have an autocorrelation in the temporal network and therefore seems to be a more dynamic construct influenced by other elements. Associations between psychological inflexibility and other constructs were limited to the between-subject level. This suggests that psychological inflexibility does not fluctuate weekly but rather mean levels of psychological inflexibility differ between individuals. Psychological inflexibility is a popular concept in clinical psychology and refers to the inadequate handling of interference or distress, the inability of taking action to manage interference or distress, avoidance behavior, and the inability of taking adaptive action that is congruent with personal goals^27^. We also found associations between psychological inflexibility, bodily weakness, and catastrophizing cognitions in the between-subject network. Average levels of psychological inflexibility were positively associated with average levels of bodily weakness, i.e., a negative self-perception of feeling weak and unable to tolerate stress and average levels of catastrophizing cognitions. Negative self-views have been found to be an important vulnerability regarding the development of clinical depression^28^ and a recent study^29^ has also found that average levels of experiential avoidance, one component of psychological inflexibility, were strongly positively associated with average levels of sadness. Importantly, patients with somatic symptoms often suffer from comorbid depression^30,31^. This points towards a potentially important role between psychological inflexibility, catastrophizing cognitions, a negative self-concept, and the co-occurrence of depression. Catastrophizing cognitions has previously been found to correlate with low coping skills^32^ which may help explain the strong association between catastrophizing cognitions and psychological inflexibility. Furthermore, we also found that weekly fluctuations of GS were associated with weekly fluctuations of stress. These associations were only present in the contemporaneous network, indicating that differences in average levels of stress are not associated with higher or lower average levels of GS. This could also point towards a potential treatment effect, as half of the patients received PMR. PMR aims to reduce stress by training individuals to distinguish between sensations of tension and relaxation, which could have weakened/eliminated the association between GS and stress. However, it may be that affect operates on a different timescale (e.g., daily or even hourly) and we were not able to catch direct associations between affect and GS due to the weekly measurements in this study. This of course not only applies to associations between elements of the affective dimension and GS but more generally to all three analyses. Finally, our findings regarding a potential causal pathway between GS and somatic distress, i.e., patient’s reaction to their throat symptoms, is in line with previous literature that found that throat symptoms are predictors of patient’s reactions^33^. However, the edge between GS and somatic distress may be of limited stability, as indicated by network stability analyses, suggesting that this edge should be interpreted with caution. Furthermore, we found a strong association between somatic distress and GS both on a within-subject and on a between-subject level in the contemporaneous and between-subject network, respectively. As such, not only did GS predict higher levels of somatic distress at the next measurement occasion, weekly fluctuations in GS were also positively associated with weekly fluctuations of somatic distress and average levels of Globus-specific symptoms were positively associated with average levels of somatic distress. Taken together, these results suggest that GS but not other behavioral related elements may have an effect on patient’s reactivity to their symptoms both short-term and long-term. Future studies could explore whether other factors may also affect somatic distress or if this construct may be influenced solely by the somatic symptoms experienced.

Our study has several strengths: First, we used data from a prospective study collected on a weekly basis during the psychotherapy period. Second, our study design not only greatly increased the strength of our evidence but also allowed the indication of a temporal sequence. Temporality is one of the nine Bradford Hill criteria^34^ which are used to provide epidemiological evidence of a causal relationship. Third, we conceptualized treatment-associated dynamics using a novel methodology in the context of psychotherapy outcome research. We illustrate how dynamic NA can be used to model therapeutic change and provide a theoretical basis for future research. Fourth, our study also assessed comorbid mental disorders, using structured clinical interviews.

The following limitations need to be considered: First, dynamic NA techniques in the context of psychology research is relatively new and as described in our Methods section, we followed the process of model selection included using significance pruning and a stepwise up model search strategy outlined in previous literature^21^. The results we reported in the manuscript and the interpretation of these results are based on the pruned models. However, discussions concerning the P value and the limitations of significance testing have recently resurfaced^35–37^ and we therefore provided all results of the baseline models in the Online Supplementary Material (Supplementary Tables S1–S9). Second, our sample size was relatively modest (*N* = 100), with on average six observations per person. This limited the number of nodes we were able to include in each analysis. Thus, we estimated separate network models and included elements related to three different dimensions: affective, cognitive, and behavioral. While this makes sense conceptually, our network models are only able to detect relationships between the variables included in each model. Hence, we were not able to model relationships between the variables of each dimension. The choice of nodes in a network is an important and influential step when it comes to the application of NA methods in psychological research, as it influences the relationships found between variables. Ideally, the chosen nodes should be able to shed light on the psychological processes the researcher wishes to examine^38^. At the same time, it is important to keep the number of nodes to a minimum in correspondence with the hypothesis of the researcher^38^. As briefly outlined above, when considering the number of nodes to include in a network, sample size / number of observations is a limiting factor. The number of nodes in a network in relation to sample size / number of observations has an impact on the stability and accuracy of networks. More nodes result in more parameters that need to be estimated and in return, greater sample sizes and/or higher numbers of observations are needed. In our case, we divided our analyses in three independent models, choosing symptoms as nodes in each analysis that correspond to three different domains of psychological experiences (affect, cognition, and behavior). We aimed to keep the number of nodes at a minimum thus limiting the number of parameters that need to be estimated in order to increase the stability and accuracy of the networks. Further studies with larger sample sizes are warranted, which would allow the inclusion of all parameters from the three domains into one analysis. Relatedly, due to sample size issues, we were not able to split our sample into subgroups (e.g., CBT versus PMR, responders versus non-responders to treatment, or patients with comorbid mental disorders versus patients without with comorbid mental disorders). Notably, network structures may differ, depending on type of received treatment, treatment response or mental comorbidity status. It would be interesting to compare different subgroups to see whether certain temporal dynamics are specific to one kind of treatment, especially considering CBT has previously been found to be the best-established treatment for a range of somatoform disorders^14^, to treatment response or to mental comorbidity status. Further studies with larger sample sizes are needed to address these questions. Third, the study design did not include a control condition beyond waiting period. Therefore, it remains to be elucidated whether the identified associations are causally linked to the treatment itself. Yet our study provides a theoretical and practical basis for future research in the field. Fourth, our model fit indices were close to appropriate, which is likely due to the low sample size and/or low number of observations per patient. Although we included only few nodes per network to compensate for the relatively low sample size, it is probable that a higher sample size and/or higher number of observations per patient would have led to better model fit indices. Fifth, timing of the assessment of the included elements can impact which associations are found since elements operate on different timescales. Our constructs were assessed weekly. On the one hand, it may make sense to measure affect more frequently as it is to be expected that affect fluctuates short-term and on the other hand it may not make sense to measure a construct such as psychological inflexibility more frequently. In fact, as our study demonstrates, psychological inflexibility appears to be relatively stable over a week and only weakly positively associated with GS short-term which is in line with previous findings^29^. Sixth, we did not record detailed information on the duration of the disease beyond recording whether the required symptom duration of at least three months with symptom onset at least six months prior to the diagnosis (part of the Rome III criteria) was fulfilled. The duration of illness may be associated with treatment response, and we would highly encourage future studies to assess duration in more detail. Seventh, all assessments were performed through self-report questionnaires. Although good concordance between self-reported medical history and medical records has been found^39^, for future studies medical records should be considered, if available. Our findings have implications for clinical research and practice. Implications for clinical research include the following: First, including nodes that operate on different timescale such as affect or psychological inflexibility likely has an impact on the associations that are found. The issue regarding analysis, interpretation, and communication of results from networks that include variables operating at different timescales is a topic that has recently been raised by Bringmann and colleagues^38^ and is currently unresolved. We encourage future studies to explore the network dynamics of disorder-relevant constructs using shorter measurement time scales, e.g., with experience-sampling methodologies for those constructs that may fluctuate on a short-term basis as well as using longer measurement time scales over longer periods of time for constructs such as dysfunctional cognitions or psychological inflexibility to examine how different measurement time scales impact findings. This may increase our understanding of the issues related to different timescales and could help establish best practices when it comes to choosing nodes and their measurement time scales in future studies. Second, by illustrating how this methodology can be used to examine the dynamics between symptoms and other relevant elements assumed to play a role in the treatment of psychological disorders, our study provides a theoretical basis for future research. In this context, future studies could investigate whether patients who respond to treatment versus those that do not, both show similar temporal dynamics. Furthermore, applying advanced methods such as experience sampling (ESM) that collect intense repeated measures of individuals, in combination with dynamic NA techniques to model therapeutic change processes, could allow the estimation of individualized networks providing insight into individual change processes related to treatment. This would be an important step towards a more personalized psychotherapy with relevant implications for clinical practice. Individualized networks may be an important and helpful research approach in accordance with the recently introduced personalized causal pathway hypothesis^40^, potentially providing an objective measure for patients’ individual symptom dynamics, which can be monitored during the psychotherapeutic process. Further, individualized networks may foster a more comprehensive view on mental health through the inclusion of elements beyond diagnostic criteria. Thus, the combination between the dynamic network approach and more specifically, its application as illustrated in this study, with repeated measures data from individuals (e.g., ESM) may be an opportunity for personalized medicine, guiding individually supported treatment decisions in line with recent literature centered around the importance of new approaches that may be able to identify individual characteristics for treatment response^41,42^. Notably, before translating the results of this work into clinical practice, further studies with larger sample sizes, including more measurement time points, as suggested by recent literature^43,44^, are required when estimating intraindividual effects; however, selected findings may hold potential clinical relevance. Our finding that catastrophizing cognitions predict bodily weakness, i.e., the negative self-concept of feeling weak and unable to tolerate stress, may have potential relevance for patients’ levels of distress and disability. As such, feeling weak or vulnerable leads to higher psychological distress^26^. Dysfunctional cognitions can be modified using psychotherapy, therefore they may represent ideal potential treatment targets in the context of GS. Therapeutic approaches targeting dysfunctional cognitions could therefore have relevance for patients’ self-concept and patients’ level of distress. Furthermore, we found positive associations between average levels of catastrophizing cognitions, psychological inflexibility, and bodily weakness. Beyond potentially leading to a more positive self-concept and lower levels of distress/disability, targeting dysfunctional cognitions could lead to higher levels of adaptive behaviors when confronted with stressful situations and perhaps even less depression symptomatology. This last point is especially relevant for patients suffering from somatic symptoms since comorbid depression is often a common feature in FSS^4^.

With regards to generalizability, our study is based on data from 100 with a common FSS, GS, that were currently undergoing group psychotherapy. We included patients across a wide range of ages, which increases generalizability. However, our study had several inclusion and exclusion criteria which decreases generalizability of our results to the general population. Furthermore, two thirds of our sample were female and a large proportion of the sample had a higher education (> 50%). These sample characteristics may decrease generalizability. Provided replicability in other indications, our findings may potentially be extended to samples diagnosed with other FSS. Additionally, we collapsed the two treatment groups into one group for reasons outlined in the limitations. It is possible that different treatments lead to different temporal and cross-sectional associations between elements, however, the current study cannot provide clarity to that end. Future studies are needed to explore treatment-specific effects. Relatedly, we would like to address the topic of replicability. Our sample size was modest and NA methods (including dynamic NA) typically require larger sample sizes for stable results. We assessed the stability of the estimated edges by performing 1000 times 25% case-drop bootstraps and found high stability in the contemporaneous networks for all three analyses, as well as in the between-subject network for the analysis examining the association between GS and cognitive elements and the analysis examining the association between GS and behavioral elements. Furthermore, we found moderate stability in the temporal networks for all three analyses and between-subject network for the analysis examining the association between GS and affective elements. It is important to note that since we already had a low sample size, dropping 25% of the sample at random and re-estimating the network model for each analysis likely influenced the stability estimates. We here report all original results and marked all stable edges, as indicated by network stability analyses. However, further studies with larger sample sizes are required before translating results of this line of work into clinical practice, preferably also from other countries and across different FSS.

To conclude, in the present study we used a systems perspective to conceptualize treatment-related temporal and cross-sectional associations of symptoms and elements related to the manifestation of a common FSS, GS, as networks of interrelated elements. GS were not predicted by any affective, cognitive, or behavioral elements related to the manifestation of GS over the course of group psychotherapy. However, our findings point towards potential treatment targets for patients with GS that may increase patient’s functioning and coping and further may have an impact on comorbid depression symptom expression. In sum, dynamic network techniques and more importantly their applications for outcome research were feasible yet are still in early stages.

## Methods

The following analyses were based on data collected in the context of a randomized clinical trial (RCT) conducted in Basel, Switzerland comparing two different types of treatment (CBT and PMR) for FSS, exemplified in patients with GS. The purpose of the RCT was to determine whether psychotherapy (based on exposure techniques and relaxation therapy, respectively) is effective in the treatment of FSS. The study was approved by the local ethics committee Ethikkomission beider Basel (EKBB) (Treatment of Globus Sensations With Psychotherapy; Reference Number Ethics Committee: 204/11). All study procedures were performed in accordance with relevant guidelines and regulations. We preregistered the RCT study on which the data analyses of this manuscript were based (Date of first registration: 03/05/2012; National Clinical Trial Identifier: NCT01590992), yet the current analyses go beyond this registration. We will report on treatment outcomes in an upcoming manuscript. Within the current manuscript, we do not adopt an RCT approach.

### Sample and procedure

Patients were recruited through Outpatient Clinics of the University Hospital Basel and through contacts with otorhinolaryngologists. Additional recruitment measures included regional postings, newspaper advertisements, and news reports. The study took place in facilities provided by the Department of Psychology of the University of Basel between May 2012 and December 2017. Patients interested in the study were first screened for study eligibility. All assessments were conducted by specifically trained assessors. Inclusion and exclusion criteria were assessed using self-report questionnaires. Inclusion criteria included age between 18 – 85 years, the ability to understand and read German, presence of Globus syndrome according to Rome III criteria^45^, and clinically significant impairment in social, occupational, or other important areas of functioning caused by GS. The majority of patients (87%) did see a medical doctor due to their throat symptoms and, if available, the Globus syndrome diagnosis was confirmed by a medical doctor. Exclusion criteria included: past 12 months severe chronic physical illnesses (especially neurological, endocrine or metabolic diseases), past month chronic medication that may interfere with the endocrine assessment, past 12 months substance dependence or eating disorder, or lifetime history of psychotic disorder or bipolar disorder. Eligible patients received information about the study and provided written informed consent in accordance with the Declaration of Helsinki. Patients were then given an appointment for the first pre-intervention assessment and randomly assigned to either the CBT or the PMR intervention.

Following the first pre-intervention, patients underwent a 1–2 month waiting period. The pre-intervention assessment took place after the waiting period. After the waiting period, the intervention took place. During the intervention phase, patients filled out online questionnaires following each treatment session. Subsequently, patients underwent a post-intervention assessment. We provide a flowchart of the study procedure in Fig. 1.

### Treatment

Treatment consisted of eight manual based 60-min group sessions of either CBT or PMR. Sessions were conducted by trained psychotherapists and took place approximately weekly in the therapeutic facilities of the Department of Psychology at the University of Basel. CBT consisted of a modified training for somatoform symptoms and was based on standardized guidelines for the psychological treatment of FSS^46^. The goals of CBT were to explain somatoform symptoms biologically and physiologically and to teach patients several coping strategies. As part of the CBT, patients were trained for self-exposure to GS which was based on a treatment manual for panic disorder with agoraphobia^47^. PMR consisted of an unspecific relaxation training and was based on a modified version of Jacobson’s original program by Bernstein and Borcove^48^ and the training manual of Hofmann^49^. For the current analyses, we combined the CBT- and the PMR-group into one intervention group.

### Intra-intervention assessment

During the weekly intervention period, patients completed online questionnaires following each treatment session, resulting in a total of 8 assessments of the following 11 constructs that have been previously linked to the manifestation of FSS^10–13^ and are therefore recommended targets in the management of FSS^10^, including affect, symptom avoidance, and beliefs and illness behavior. Positive affect (PosA) and negative affect (NegA) was assessed using the Positive and Negative Affect Scale-last day (PANAS-D)^50,51^ and 10 NegA items. The PANAS is considered to be reliable and valid measure for the two dimensions of mood, PosA and NegA, across different subject populations and different time frame^50^. Cronbach’s alphas range from 0.84 (NegA) to 0.90 (PosA)^52^. The scale ranged from 0 = Very slightly or Not at all to 4 = Extremely. Stress was assessed with one item rated on an 11-point scale ranging from 0 = Not at all to 10 = Extremely from the Daily Symptom Exposure Questionnaire (DSE-Q). Psychological inflexibility, which includes the component experiential avoidance, was assessed using the Acceptance and Action Questionnaire (AAQ-II)^53^ which includes 10 items each rated on an 8-point scale ranging from 1 = Never true to 7 = Always true. The AAQ-II has a very good level or reliability with an average Cronbach’s alpha of 0.84^53^. The questionnaire was scored by adding up the responses for each item returning values between 10 and 70. Lower total scores indicate higher flexibility while higher total scores indicate lower flexibility. The Cognitions About Body and Health Questionnaire (CABAH)^12,13^ was used to assess specific dysfunctional cognitions and behaviors related to the development of somatoform disorders. Cronbach’s alpha for the CABAH questionnaire ranges from 0.6 (for the factor health habits) to 0.89 (for the factor catastrophizing cognitions) in clinical samples^12^. The items used to measure these cognitions/behaviors were rated on a 4-point scale from 3 = Completely right to 0 = Completely wrong: Catastrophizing cognitions (the scoring returned values between 0 and 42, i.e., low, respectively high tendency to rate body signals in a catastrophizing manner); bodily weakness (the scoring returned values between 0 and 18, i.e., low, respectively high tendency for a negative self-concept of being weak, feeling exhausted and not able to tolerate stress); intolerance of bodily complaints (the scoring returned values between 0 and 12, i.e., low, respectively high tendency for the inability to tolerate general bodily complaints; importantly, this construct has high relevance for behaviors related to health care utilization as bodily complaints cause these individuals to seek help immediately^4^); health habits (the scoring returned values between 0 and 9, i.e., low, respectively high tendency for typical habits related to people wanting to live a healthy lifestyle). Finally, the Glasgow Edinburgh Throat Scale (GETS)^54^ is a 12 item questionnaire that was used to assess throat symptoms as well as patients’ reactions to their throat symptoms. The GETS has been validated for use in patients with GS and Cronbach’s alpha of 0.83^55^. The items were rated on an 8-point scale from 0 = None/Not at all to 7 = Unbearable/All the time/Extremely: GS was assessed by three items that previously showed very high loadings on a factor specific to Globus: “Feeling of something stuck in the throat”, “Discomfort/irritation in the throat”, and “Want to swallow all the time”; Somatic distress was assessed by the following two items: “How much time do you spend thinking about your throat?” and “At present, how annoying do you find your throat sensation?”. We standardized all variables.

### Statistical analyses

We conducted three separate network analyses to capture temporal and cross-sectional associations between key elements related to the (1) affective, (2) cognitive, and (3) behavioral dimension over the course of treatment. In the first analysis, we examined the relationships between PosA, NegA, stress, and GS. In the second analysis, we examined the relationships between dysfunctional cognitions related to the manifestation of GS (psychological inflexibility, catastrophizing cognitions, and bodily weakness) and GS. In the third analysis, we examined the relationships between behavioral related elements (intolerance of bodily complaints, health habits, and somatic distress) and GS. For each analysis we estimated a multilevel lag-1 dynamic network model using the ml_ts_lvgvar function implemented in the R-package psychonetrics^21^. We accounted for missing data by using the full-information maximum likelihood estimation. We pruned the resulting baseline models for all three analyses using an alpha level of 0.05, which reduced model complexity by removing non-significant associations. We further used a step-up model search to optimize the Bayesian information criterion (BIC). We compared the baseline model, the pruned model, and the step-up model for each separate analysis based on the root mean square error of approximation (RMSEA), BIC, and the Akaike information criterion (AIC) and chose the model with the best fit. We then computed the following model fit statistics: the comparative fit index (CFI), Tucker-Lewis index (TLI), and RMSEA. Each model resulted in three networks, each offering different information: (1) a temporal network, in which each variable at timepoint t is regressed on all other variables at t + 1 describing how each variable predicts all other variables at the next measurement occasion; (2) a within-subject contemporaneous network, which represents concurrent associations between variables while adjusting for temporal associations; and (3) a between-subject network, which represents the relationship between the means of variables over all measurement occasions while adjusting for all other variables in the network. Importantly, between-person mean differences modelled in the between-subject network provide more information regarding long-term interventions than the contemporaneous network, which models short-term and fleeting associations^38^. Finally, to examine the stability of the estimated network parameters, we conducted network stability estimation, following established procedures^21,56^. To this end, we estimated 1000 25% case-drop bootstrapped samples and re-estimated the network structure of each sample using the respective search strategies applied in the main analyses. We then assessed the level of stability by examining the frequency of each edge being included in the estimated models of the 1000 bootstrap samples. Edges that are included in at least 50% of the bootstrapped samples were considered stable^57^.

For reasons of completeness and transparency, we provide all results irrespective of the conventional significance level of the baseline models in the Online Supplementary Material. Data were analyzed and estimated networks were visualized using R, version 4.1.0^58^ in R-Studio 1.4.1717-3 and the R-package qgraph^59^. We performed all calculations at sciCORE (http://scicore.unibas.ch) scientific computing center at the University of Basel.

### Supplementary Information

Supplementary Information.

## Data Availability

The following analyses were based on data from a registered randomized controlled trial (RCT; National Clinical Trial Identifier: NCT01590992). The data and material of the study are not publicly available due to privacy or ethical restrictions. In the case of inquiries by third parties that wish to reuse data that support the findings of this manuscript, they may submit a request to the corresponding author and will have to obtain authorization of the responsible ethics committee as ordained in the Ordinance of 20 September 2013 on Human Research with the exception of Clinical Trials (Human Research Ordinance, HRO). Only upon collection of all important consents and upon approval of the responsible ethics committee(s), the fully anonymized requested data will be transferred to the third party. Third parties must confirm and provide evidence to comply with all relevant Swiss and cantonal laws and regulations (especially regarding data protection and Human Research), as well as all obligations and regulations set out in the documents and contracts related to the project. Fees may apply to cover expenses related to the data reuse. The clinical trial protocol, intervention manuals, as well as the R-code for the statistical analysis are available upon request.
