# Supplementary material for: Treatment-associated network dynamics in patients with globus sensations: a proof-of-concept study
Source: Sci Rep. 2023 Sep 20;13:15615. doi: 10.1038/s41598-023-42186-y (PMC10511470; doi:10.1038/s41598-023-42186-y)
Supplement: Supplementary file 1 — Supplementary Information. [file 41598_2023_42186_MOESM1_ESM.docx]

**Online Supplementary Material for:**

**“Treatment-Associated Network Dynamics in Patients With Globus Sensations: A Proof-of-Concept Study”**

Marina N. Imperiale, Roselind Lieb, and Gunther Meinlschmidt*

**Table S1-S9**: All results, irrespective of the conventional significance level of the baseline models.

**Table S10-S12**: Results of the 25% case-dropped bootstrap samples

**Table S1**

*Temporal Network Associations of the Baseline Model for Analysis 1: Associations Between Affective Elements and GS*

| Edge | Estimate | 95% CI | | *p* |
| --- | --- | --- | --- | --- |
|  |  | *LL* | *UL* |  |
| Positive Affect -> Positive Affect | .190 | .075 | .305 | .001 |
| Positive Affect -> Negative Affect | .027 | -.097 | .150 | .674 |
| Positive Affect -> Stress | .015 | -.130 | .160 | .842 |
| Positive Affect -> Globus Sensations | .028 | -.091 | .148 | .641 |
| Negative Affect -> Positive Affect | -.036 | -.130 | .057 | .445 |
| Negative Affect -> Negative Affect | .150 | .032 | .269 | .013 |
| Negative Affect -> Stress | -.135 | -.263 | -.006 | .039 |
| Negative Affect -> Globus Sensations | 0 | -.105 | .106 | .994 |
| Stress -> Positive Affect | -.018 | -.097 | .060 | .644 |
| Stress -> Negative Affect | .007 | -.085 | .099 | .883 |
| Stress -> Stress | .332 | .214 | .449 | <.001 |
| Stress -> Globus Sensations | -.040 | -.129 | .049 | .377 |
| Globus Sensations -> Positive Affect | 0 | -.090 | .090 | .999 |
| Globus Sensations -> Negative Affect | .057 | -.048 | .162 | .288 |
| Globus Sensations -> Stress | -.113 | -.236 | .010 | .072 |
| Globus Sensations -> Globus Sensations | .354 | .240 | .467 | <.001 |

*Note*. CI: Confidence Interval. *LL*: Lower Limit. *UL*: Upper Limit

**Table S2**

*Contemporaneous Network Associations of the Baseline Model for Analysis 1: Associations Between Affective Elements and GS*

| Edge | Estimate | 95% CI | | *p* |
| --- | --- | --- | --- | --- |
|  |  | *LL* | *UL* |  |
| Negative Affect -- Positive Affect | -.131 | -.219 | -.044 | .003 |
| Stress -- Positive Affect | -.163 | -.25 | -.077 | <.001 |
| Stress -- Negative Affect | .306 | .226 | .387 | <.001 |
| Globus Sensations -- Positive Affect | .028 | -.061 | .117 | .543 |
| Globus Sensations -- Negative Affect | .048 | -.041 | .137 | .289 |
| Globus Sensations -- Stress | .130 | .043 | .217 | .003 |

*Note.* CI: Confidence Interval. *LL*: Lower Limit. *UL*: Upper Limit.

**Table S3**

*Between-Subject Network Associations of the Baseline Model for Analysis 1: Associations Between Affective Elements and GS*

| Edge | Estimate | 95% CI | | *p* |
| --- | --- | --- | --- | --- |
|  |  | *LL* | *UL* |  |
| Negative Affect -- Positive Affect | -.303 | -.524 | -.083 | .007 |
| Stress -- Positive Affect | -.314 | -.556 | -.071 | .011 |
| Stress -- Negative Affect | .327 | .080 | .573 | .009 |
| Globus Sensations -- Positive Affect | .189 | -.044 | .422 | .113 |
| Globus Sensations -- Negative Affect | -.026 | -.275 | .223 | .838 |
| Globus Sensations -- Stress | .188 | -.085 | .461 | .177 |

*Note.* CI: Confidence Interval. *LL*: Lower Limit. *UL*: Upper Limit.

**Table S4**

*Temporal Network Associations of the Baseline Model for Analysis 2: Associations Between Cognitive Elements and GS*

| Edge | Estimate | 95% CI | | *p* |
| --- | --- | --- | --- | --- |
|  |  | *LL* | *UL* |  |
| Psychological Inflexibility -> Psychological Inflexibility | .130 | .018 | .242 | .023 |
| Psychological Inflexibility -> Catastrophizing Cognitions | .092 | -.004 | .188 | .062 |
| Psychological Inflexibility -> Bodily Weakness | .068 | -.023 | .158 | .142 |
| Psychological Inflexibility -> Globus Sensations | .026 | -.125 | .176 | .739 |
| Catastrophizing Cognitions -> Psychological Inflexibility | .078 | -.035 | .191 | .175 |
| Catastrophizing Cognitions -> Catastrophizing Cognitions | .262 | .147 | .378 | <.001 |
| Catastrophizing Cognitions -> Bodily Weakness | .092 | -.006 | .192 | .067 |
| Catastrophizing Cognitions -> Globus Sensations | -.033 | -.198 | .132 | .697 |
| Bodily Weakness -> Psychological Inflexibility | .115 | -.005 | .235 | .059 |
| Bodily Weakness -> Catastrophizing Cognitions | -.044 | -.156 | .069 | .447 |
| Bodily Weakness -> Bodily Weakness | .074 | -.039 | .188 | .200 |
| Bodily Weakness -> Globus Sensations | .136 | -.040 | .311 | .130 |
| Globus Sensations -> Psychological Inflexibility | .046 | -.023 | .116 | .191 |
| Globus Sensations -> Catastrophizing Cognitions | .032 | -.033 | .097 | .328 |
| Globus Sensations -> Bodily Weakness | .048 | -.013 | .109 | .119 |
| Globus Sensations -> Globus Sensations | .353 | .240 | .466 | <.001 |

*Note.* CI: Confidence Interval. *LL*: Lower Limit. *UL*: Upper Limit.

**Table S5**

*Contemporaneous Network Associations of the Baseline Model for Analysis 2: Associations Between Cognitive Elements and GS*

| Edge | Estimate | 95% CI | | *p* |
| --- | --- | --- | --- | --- |
|  |  | *LL* | *UL* |  |
| Catastrophizing Cognitions -- Psychological Inflexibility | .056 | -.032 | .145 | .213 |
| Bodily Weakness -- Psychological Inflexibility | .076 | -.013 | .164 | .094 |
| Globus Sensations -- Psychological Inflexibility | .110 | .018 | .196 | .018 |
| Bodily Weakness -- Catastrophizing Cognitions | .242 | .158 | .326 | <.001 |
| Globus Sensations -- Catastrophizing Cognitions | .077 | -.011 | .166 | .087 |
| Globus Sensations -- Bodily Weakness | -.018 | -.110 | .072 | .692 |

*Note.* CI: Confidence Interval. *LL*: Lower Limit. *UL*: Upper Limit.

**Table S6**

*Between-Subject Network Associations of the Baseline Model for Analysis 2: Associations Between Cognitive Elements and GS*

| Edge | Estimate | 95% CI | | *p* |
| --- | --- | --- | --- | --- |
|  |  | *LL* | *UL* |  |
| Catastrophizing Cognitions -- Psychological Inflexibility | .292 | .098 | .486 | .032 |
| Bodily Weakness -- Psychological Inflexibility | .420 | .246 | .593 | <.001 |
| Globus Sensations -- Psychological Inflexibility | -.105 | -.331 | .121 | .362 |
| Bodily Weakness -- Catastrophizing Cognitions | .328 | .140 | .516 | <.001 |
| Globus Sensations -- Catastrophizing Cognitions | .049 | -.178 | .275 | .674 |
| Globus Sensations -- Bodily Weakness | .127 | -.096 | .349 | .264 |

*Note.* CI: Confidence Interval. *LL*: Lower Limit. *UL*: Upper Limit.

**Table S7**

*Temporal Network Associations of the Baseline Model for Analysis 3: Associations Between Behavioral Elements and GS*

| Edge | Estimate | 95% CI | | *p* |
| --- | --- | --- | --- | --- |
|  |  | *LL* | *UL* |  |
| Intolerance of Bodily Complaints -> Intolerance of Bodily Complaints | .135 | .023 | .246 | .018 |
| Intolerance of Bodily Complaints -> Health Habits | .037 | -.053 | .127 | .419 |
| Intolerance of Bodily Complaints -> Somatic Distress | .109 | -.035 | .253 | .139 |
| Intolerance of Bodily Complaints -> Globus Sensations | -.025 | -.152 | .101 | .696 |
| Health Habits -> Intolerance of Bodily Complaints | -.063 | -.180 | .053 | .284 |
| Health Habits -> Health Habits | .157 | .046 | .268 | .006 |
| Health Habits -> Somatic Distress | -.025 | -.189 | .139 | .763 |
| Health Habits -> Globus Sensations | .045 | -.099 | .189 | .539 |
| Somatic Distress -> Intolerance of Bodily Complaints | .059 | -.034 | .152 | .213 |
| Somatic Distress -> Health Habits | -.072 | -.154 | .009 | .083 |
| Somatic Distress -> Somatic Distress | .173 | .033 | .313 | .015 |
| Somatic Distress -> Globus Sensations | -.004 | -.119 | .111 | .950 |
| Globus Sensations -> Intolerance of Bodily Complaints | -.041 | -.148 | .066 | .451 |
| Globus Sensations -> Health Habits | .043 | -.050 | .137 | .367 |
| Globus Sensations -> Somatic Distress | .196 | .045 | .346 | .011 |
| Globus Sensations -> Globus Sensations | .353 | .213 | .493 | <.001 |

*Note.* CI: Confidence Interval. *LL*: Lower Limit. *UL*: Upper Limit.

**Table S8**

*Contemporaneous Network Associations of the Baseline Model for Analysis 3: Associations Between Behavioral Elements and GS*

| Edge | Estimate | 95% CI | | *p* |
| --- | --- | --- | --- | --- |
|  |  | *LL* | *UL* |  |
| Health Habits -- Intolerance of Bodily Complaints | -.012 | -.101 | .077 | .789 |
| Somatic Distress -- Intolerance of Bodily Complaints | .004 | -.085 | .093 | .931 |
| Globus Sensations -- Intolerance of Bodily Complaints | -.050 | -.139 | .039 | .267 |
| Somatic Distress -- Health Habits | .020 | -.069 | .109 | .660 |
| Globus Sensations -- Health Habits | -.015 | -.104 | .074 | .745 |
| Globus Sensations -- Somatic Distress | .641 | .588 | .693 | <.001 |

*Note.* CI: Confidence Interval. *LL*: Lower Limit. *UL*: Upper Limit.

**Table S9**

*Between-Subject Network Associations of the Baseline Model for Analysis 3: Associations Between Behavioral Elements and GS*

| Edge | Estimate | 95% CI | | *p* |
| --- | --- | --- | --- | --- |
|  |  | *LL* | *UL* |  |
| Health Habits -- Intolerance of Bodily Complaints | -.144 | -.353 | .065 | .177 |
| Somatic Distress -- Intolerance of Bodily Complaints | .333 | .123 | .542 | .002 |
| Globus Sensations -- Intolerance of Bodily Complaints | -.265 | -.477 | -.053 | .014 |
| Somatic Distress -- Health Habits | .052 | -.182 | .287 | .661 |
| Globus Sensations -- Health Habits | -.061 | -.285 | .164 | .597 |
| Globus Sensations -- Somatic Distress | .713 | .589 | .838 | <.001 |

*Note.* CI: Confidence Interval. *LL*: Lower Limit. *UL*: Upper Limit.

**Table S10**

*Frequency of Edge Inclusion in 1000 Iterations of 25% Case-Dropped Bootstrapped Samples for Analysis 1: Associations Between Affective Elements and GS*

| Edge | Frequency |
| --- | --- |
| Temporal Associations |  |
| **Positive Affect -> Positive Affect** | 458* |
| Positive Affect -> Negative Affect | 7 |
| Positive Affect -> Stress | 2 |
| Positive Affect -> Globus Sensations | 9 |
| Negative Affect -> Positive Affect | 7 |
| **Negative Affect -> Negative Affect** | 282* |
| Negative Affect -> Stress | 140 |
| Negative Affect -> Globus Sensations | 8 |
| Stress -> Positive Affect | 3 |
| Stress -> Negative Affect | 1 |
| **Stress -> Stress** | 982 |
| Stress -> Globus Sensations | 13 |
| Globus Sensations -> Positive Affect | 8 |
| Globus Sensations -> Negative Affect | 16 |
| Globus Sensations -> Stress | 100 |
| **Globus Sensations -> Globus Sensations** | 981 |
| Contemporaneous Associations | |
| **Negative Affect -- Positive Affect** | 502 |
| **Stress -- Positive Affect** | 812 |
| **Stress -- Negative Affect** | 1000 |
| Globus Sensations -- Positive Affect | 1 |
| Globus Sensations -- Negative Affect | 25 |
| **Globus Sensations -- Stress** | 505 |
| Between-Subject Associations | |
| **Negative Affect -- Positive Affect** | 644 |
| **Stress -- Positive Affect** | 317* |
| **Stress -- Negative Affect** | 447* |
| Globus Sensations -- Positive Affect | 4 |
| Globus Sensations -- Negative Affect | 0 |
| Globus Sensations -- Stress | 4 |

*Note.* This table shows all possible edges. Edges that were found in 50% or more of bootstrapped samples (i.e., frequency >= 500) are considered robust. Significant edges (< .05) are bold. *indicates non-robust edges that were found and reported in the original analyses.

**Table S11**

| Edge | Frequency |
| --- | --- |
| Temporal Associations |  |
| **Psychological Inflexibility -> Psychological Inflexibility** | 327* |
| Psychological Inflexibility -> Catastrophizing Cognitions | 115 |
| Psychological Inflexibility -> Bodily Weakness | 50 |
| Psychological Inflexibility -> Globus Sensations | 3 |
| Catastrophizing Cognitions -> Psychological Inflexibility | 62 |
| **Catastrophizing Cognitions -> Catastrophizing Cognitions** | 875 |
| **Catastrophizing Cognitions -> Bodily Weakness** | 297* |
| Catastrophizing Cognitions -> Globus Sensations | 4 |
| Bodily Weakness -> Psychological Inflexibility | 72 |
| Bodily Weakness -> Catastrophizing Cognitions | 12 |
| Bodily Weakness -> Bodily Weakness | 112 |
| Bodily Weakness -> Globus Sensations | 52 |
| Globus Sensations -> Psychological Inflexibility | 23 |
| Globus Sensations -> Catastrophizing Cognitions | 17 |
| Globus Sensations -> Bodily Weakness | 22 |
| **Globus Sensations -> Globus Sensations** | 986 |
| Contemporaneous Associations | |
| Catastrophizing Cognitions -- Psychological Inflexibility | 23 |
| Bodily Weakness -- Psychological Inflexibility | 69 |
| **Bodily Weakness -- Catastrophizing Cognitions** | 997 |
| Globus Sensations -- Psychological Inflexibility | 236 |
| Globus Sensations -- Catastrophizing Cognitions | 44 |
| Globus Sensations -- Bodily Weakness | 0 |
| Between-Subject Associations | |
| **Catastrophizing Cognitions -- Psychological Inflexibility** | 668 |
| **Bodily Weakness -- Psychological Inflexibility** | 1000 |
| **Bodily Weakness -- Catastrophizing Cognitions** | 999 |
| Globus Sensations -- Psychological Inflexibility | 1 |
| Globus Sensations -- Catastrophizing Cognitions | 1 |
| Globus Sensations -- Bodily Weakness | 1 |

*Frequency of Edge Inclusion in 1000 Iterations of 25% Case-Dropped Bootstrapped Samples for Analysis 2: Associations Between Cognitive Elements and GS*

*Note.* This table shows all possible edges. Edges that were found in 50% or more of bootstrapped samples (i.e., frequency >= 500) are considered robust. Significant edges (< .05) are bold. *indicates non-robust edges that were found and reported in the original analyses.

**Table S12**

*Frequency of Edge Inclusion in 1000 Iterations of 25% Case-Dropped Bootstrapped Samples for Analysis 3: Associations Between Behavioral Elements and GS*

| Edge | Frequency |
| --- | --- |
| Temporal Associations |  |
| Intolerance of Bodily Complaints -> Intolerance of Bodily Complaints | 227 |
| Intolerance of Bodily Complaints -> Health Habits | 42 |
| Intolerance of Bodily Complaints -> Somatic Distress | 84 |
| Intolerance of Bodily Complaints -> Globus Sensations | 6 |
| Health Habits -> Intolerance of Bodily Complaints | 14 |
| **Health Habits -> Health Habits** | 354* |
| Health Habits -> Somatic Distress | 6 |
| Health Habits -> Globus Sensations | 13 |
| Somatic Distress -> Intolerance of Bodily Complaints | 16 |
| Somatic Distress -> Health Habits | 91 |
| **Somatic Distress -> Somatic Distress** | 536 |
| Somatic Distress -> Globus Sensations | 11 |
| Globus Sensations -> Intolerance of Bodily Complaints | 11 |
| Globus Sensations -> Health Habits | 27 |
| **Globus Sensations -> Somatic Distress** | 432* |
| **Globus Sensations -> Globus Sensations** | 921 |
| Contemporaneous Associations | |
| Health Habits -- Intolerance of Bodily Complaints | 0 |
| Somatic Distress -- Intolerance of Bodily Complaints | 0 |
| Globus Sensations -- Intolerance of Bodily Complaints | 11 |
| Somatic Distress -- Health Habits | 4 |
| Globus Sensations -- Health Habits | 0 |
| **Globus Sensations -- Somatic Distress** | 1000 |
| Between-Subject Associations | |
| Health Habits -- Intolerance of Bodily Complaints | 0 |
| **Somatic Distress -- Intolerance of Bodily Complaints** | 845 |
| Globus Sensations -- Intolerance of Bodily Complaints | 395 |
| Somatic Distress -- Health Habits | 0 |
| Globus Sensations -- Health Habits | 0 |
| **Globus Sensations -- Somatic Distress** | 999 |

*Note.* This table shows all possible edges. Edges that were found in 50% or more of bootstrapped samples (i.e., frequency >= 500) are considered robust. Significant edges (< .05) are bold. *indicates non-robust edges that were found and reported in the original analyses.
